# Digital Oral Medicine for the Elderly

**DOI:** 10.3390/ijerph17072171

**Published:** 2020-03-25

**Authors:** Christian E. Besimo, Nicola U. Zitzmann, Tim Joda

**Affiliations:** Department of Reconstructive Dentistry, University Center for Dental Medicine Basel, University of Basel, 4058 Basel, Switzerland; n.zitzmann@unibas.ch (N.U.Z.); tim.joda@unibas.ch (T.J.)

**Keywords:** oral medicine, oral healthcare, dentistry, gerodontology, elderly patient, digital transformation, big data, patient-centered outcomes

## Abstract

Sustainable oral care of the elderly requires a holistic view of aging, which must extend far beyond the narrow field of dental expertise to help reduce the effects of sociobiological changes on oral health in good time. Digital technologies now extend into all aspects of daily life. This review summarizes the diverse digital opportunities that may help address the complex challenges in Gerodontology. Systemic patient management is at the center of these descriptions, while the application of digital tools for purely dental treatment protocols is deliberately avoided.

## 1. Introduction

The steady aging of human populations is a development that affects not only the industrialized world, but also emerging and developing countries. It is estimated that about half of all people who have ever lived to an age of 65 years old or older are alive today. We are living through an exponential population expansion and demographic transition. Therefore, it is necessary to understand the sociological and biological changes facing the elderly population and to master the current and future challenges in dental healthcare for aging patients [1].

This opinion letter, based on an ongoing evaluation of the sociodemographic changes due to aging, focuses on digital technologies, which could help deal with the complex challenges in oral medicine for the growing elderly.

## 2. A Silent Revolution

### 2.1. Social Change

Old age is changing fundamentally and to an extent that justifies the term ‘social revolution’, albeit one that is proceeding quietly. This change is characterized by the objective of being able to live in a self-determined manner and in a private environment for as long as possible, even when in need of healthcare. In this context, a transfer to a care institution is only foreseen in the case of an extreme emergency and to be delayed for as long as possible. This development will contribute to the progressive delaying of the fourth age, which is marked by the need for advanced assistance and care, and will further reduce the average length of stay in institutions. In Switzerland, individuals aged 65 years and older only stay in nursing homes for one year [2].

It is important to recognize the goal-oriented willingness and the high degree of creativity that senior citizens, either currently working or retired, display in their third age (traditionally 65–80 years old). However, these factors do not allow for any reliable prognoses regarding changes in lifestyles in old age and force the professional groups, institutions, and organizations concerned with aging to continually adapt their strategies and concepts [2]. This awareness has also reached the political arena in Switzerland, so that in future, there will be a growing reluctance to plan new inpatient care places and priority will be given to outpatient care in terms of cost-effectiveness [3,4].

### 2.2. Consequences for Health

The biological limit for life expectancy at birth and after reaching the age of 65 is still not predictable. Medical advances, healthy nutrition, good education, and improving working conditions continue to favor an increasingly longer third age and will reduce the risk and duration of the fourth age [2].

The preventive and restorative success of dentistry have led to people with an increasing number of teeth (including implant-supported reconstructions). However, despite their knowledge of the importance of regular dental check-ups for oral and general health, the elderly will inevitably gradually withdraw from this care, beginning between the ages of 60 and 65 [5]. The risk of psychosocial (loneliness, poverty) and medical problems (multimorbidity, polypharmacy), which increase with age, play a central role in withdrawing from care with major consequences for dental and oral health in the long term. Oral diseases do not only occur in old age when the need for help and care begins, but much earlier, because the social and biological factors mentioned above increasingly affect the resources needed to maintain oral hygiene and to receive regular care from the personal dental team. Even if the fourth age is delayed, the oral health issues still inevitably arise, and are then complicated further by the additional comorbidities of aging [6].

Facing these complex challenges, it is important for dentists to learn to perceive the human being holistically—in her or his entirety—and to establish a close network with other medical disciplines, institutions, organizations, authorities, and relatives who are concerned with the care of aging people. It is important to be aware that the range of stakeholders involved is growing and becoming more volatile, as the shift from inpatient to outpatient care increases [7,8].

## 3. Digital Opportunities

People participating in the digital community generate a rapidly growing amount of data every day. This is also increasingly true for senior citizens. Scientific use of this data offers the opportunity to gain a deeper and more dynamic insight into the lifestyle of aging people, for example, through analyzing digital shopping activities and payment transactions. This could allow a better and more up-to-date understanding of the changing lifestyles of the elderly. It is conceivable that algorithms could be developed that can identify sociobiological threats at an early stage by monitoring changes in behavior. Such algorithms would also be important for the dental care of aging people and thus for oral health. This would be one of several opportunities to achieve a paradigm shift in geriatric dentistry and to promote preventive rather than palliative care concepts that are still predominant [9,10].

### 3.1. In Frigo Veritas (The Truth Lies in the Fridge)

The *“In Frigo Veritas*” study conducted in Geneva in the 1990s demonstrated that the contents of the refrigerators of senior citizens was associated with the likelihood of hospitalization in the following month (11). Monitoring the nutritional provisions available to an elderly individual could therefore identify those at risk early. The use of shopping lists of food products, which are already electronically recorded today with the help of customer cards, could be considered here. This data alone would already allow individual conclusions to be drawn about the quantity, quality, and course of food. A link to intelligent refrigerator systems that can document the consumption and replenishment of food would also be conceivable. This would allow continuous conclusions to be drawn in real time on the nutritional situation and thus the morbidity risk in an out-of-home care setting [11]. This application could also be used in dentistry for therapeutic decision making or for the ongoing assessment of the care capacity of aging people threatened by sociobiological risks. In addition, nutritional counselling and guidance, supported by nutritional algorithms, could be carried out in a simplified, individualized and continuous manner, before, during, and/or after dental interventions such as tooth extractions or the insertion of fixed and removable dentures [12].

### 3.2. Intelligent, Individually Usable Systems

The personal health data generated in medicine, including dentistry, or by intelligent systems suitable for everyday use, such as smartphones, watches or other devices, open up a wide range of application options that will go far beyond the recording of acute emergency situations in in-home and out-of-home care settings. On the one hand, the cumulative use of medically relevant data does not only offer significantly expanded perspectives for research, but also for patient care. Today, it is already feasible to record vital data in real time using the aforementioned intelligent everyday systems. It can be assumed that the availability and variety of such systems will continuously increase in the near future and will also be usefully applied in dentistry [13,14].

### 3.3. Stop Walking When Talking

Nowadays, electronic pedometers are used to obtain discounts from health insurance companies. Similarly, we are already able to analyze gait regularity and thus the risk of falls among older people in specialized mobility centers, with or without multitasking, and to draw conclusions about diseases, side effects of medication, and cognitive performance [15]. The transfer of such systems to shoe insoles, for example, not only has the potential to obtain and link incomparably more empirical data on gait safety in elderly people living in a private household, but also to monitor their mobility in real time. In this context, the effects of therapeutic interventions on gait safety, such as those that aim to optimize occlusion, could be dynamically monitored [16].

## 4. Interdisciplinary Networking

As mentioned previously, the (dental) medical care of aging people living in private households is faced with growing interdisciplinary challenges. On the one hand, healthcare providers have to establish a network to harness the knowledge of the various disciplines by means of suitable digital systems to make it not only accessible for interdisciplinary research, but also clinically usable under growing organizational and legal requirements. On the other hand, everyday clinical practice requires dynamic, real-time networking among the growing number of stakeholders in the care of the elderly, which will increase significantly and become more volatile as outpatient care expands. Here, intelligent tools are needed that enable compatible, rapid, and secure interdisciplinary data exchange on a patient-by-patient basis to support individually tailored decision-making based on algorithms [17].

Finally, it is expected that routine sequencing of the genome in the case of disease will become established within the next five to ten years, as the costs of this procedure have been significantly reduced from $100,000 to $1000 over the last 20 years [18]. This should also contribute to the individualization of prevention, diagnostics, and therapy in dentistry, especially for older people with increasing psychosocial and medical risks. The latter could possibly be detected earlier and counteracted more effectively [19].

## 5. Ethical and Legal Responsibilities

We have learned from the hitherto short history of the digitalization of our world that this development is accelerating at a breathtaking rate. This calls for an urgent and internationally valid regulation for the protection of personal data of individuals, while enabling the exchange of personal information between stakeholders for the benefit of the individual. This has been pioneered by the basic data protection regulation of the European Union [20]. Such a set of rules must compensate for the existing socio-economic asymmetry of a data-driven economy, which ensures the right to a copy of personal data and thus digital self-determination. However, the right to a copy of personal data also requires the development of cooperatively managed databases that are able to manage the digital information in a fiduciary capacity and in a comparable way to financial institutions. In this way, it would be ensured that people could come into possession of all their health-related data to use these under regulated conditions for their own benefit or to make data available to research and thus to the community [21].

In addition, society must ensure that (dental) medicine, which is increasingly controlled by guidelines and algorithms, does not lose sight of the individual person. It is true that large amounts of data can increase the reliability of answers to individual questions. Nevertheless, it remains to be hoped that big data will not lead to further commercialization or industrialization of medicine, and thus, neglect the healing power of a systemic doctor-patient relationship, but rather that it will nurture this relationship [22,23].

## 6. Conclusions

The global demographic change is characterized by an exponential population expansion and sociobiological transition towards a growing number of older patients. Sustainable oral healthcare of the elderly must comprise a holistic view of aging, far beyond the narrow field of dental diagnostics and modernized treatment protocols. Digital health data generated in dental medicine, or by daily used systems, such as smartphones, tablets, and watches, open up a wide range of application options in (oral) healthcare to master the complex challenges in Gerodontology. Scientific use of this data offers broad insights into the lifestyle of aging patients for the early identification of social threats and changing behaviors.

Medical and dental healthcare providers have to establish an interdisciplinary network using these digital systems for routine clinical practice. Smart digital applications are needed, which enable compatible, rapid, and secure interdisciplinary data exchange on a patient-by-patient level to support individually tailored decision-making based on the knowledge of all stakeholders in the care of the elderly in in-home and out-of-home care settings. The digital transformation has the opportunity to achieve a paradigm shift in geriatric dentistry and to promote preventive rather than palliative healthcare concepts.
